# Pyrazinoic acid, the active form of the anti-tuberculosis drug pyrazinamide, and aromatic carboxylic acid analogs are protonophores

**DOI:** 10.3389/fmolb.2024.1350699

**Published:** 2024-02-13

**Authors:** Fabio L. Fontes, Steven A. Rooker, Jamie K. Lynn-Barbe, Michael A. Lyons, Debbie C. Crans, Dean C. Crick

**Affiliations:** ^1^ Program in Cell and Molecular Biology, Colorado State University, Fort Collins, CO, United States; ^2^ Mycobacteria Research Laboratories, Department of Microbiology, Immunology and Pathology, Colorado State University, Fort Collins, CO, United States; ^3^ Department of Chemistry, Colorado State University, Fort Collins, CO, United States

**Keywords:** uncoupling, QSAR, growth inhibition, *Mycobacterium tuberculosis*, benzoic acid, salicylic acid, *para*-aminosalicylic acid, picolinic acid

## Abstract

Pyrazinoic acid is the active form of pyrazinamide, a first-line antibiotic used to treat *Mycobacterium tuberculosis* infections. However, the mechanism of action of pyrazinoic acid remains a subject of debate, and alternatives to pyrazinamide in cases of resistance are not available. The work presented here demonstrates that pyrazinoic acid and known protonophores including salicylic acid, benzoic acid, and carbonyl cyanide *m*-chlorophenyl hydrazone all exhibit pH-dependent inhibition of mycobacterial growth activity over a physiologically relevant range of pH values. Other anti-tubercular drugs, including rifampin, isoniazid, bedaquiline, and *p*-aminosalicylic acid, do not exhibit similar pH-dependent growth-inhibitory activities. The growth inhibition curves of pyrazinoic, salicylic, benzoic, and picolinic acids, as well as carbonyl cyanide *m*-chlorophenyl hydrazone, all fit a quantitative structure–activity relationship (QSAR) derived from acid–base equilibria with R^2^ values > 0.95. The QSAR model indicates that growth inhibition relies solely on the concentration of the protonated forms of these weak acids (rather than the deprotonated forms). Moreover, pyrazinoic acid, salicylic acid, and carbonyl cyanide *m*-chlorophenyl hydrazone all caused acidification of the mycobacterial cytoplasm at concentrations that inhibit bacterial growth. Thus, it is concluded that pyrazinoic acid acts as an uncoupler of oxidative phosphorylation and that disruption of proton motive force is the primary mechanism of action of pyrazinoic acid rather than the inhibition of a classic enzyme activity.

## Introduction

Pyrazinoic acid (POA) is the active form of the prodrug pyrazinamide (PZA), an antibiotic used in combination with other drugs in standard treatment regimens for tuberculosis (TB) infections (World Health Organization, 2018). Multiple reports indicate that the addition of PZA to the various regimens available for TB treatment reduced the average time of treatment from 9–12 months to 6 months (British Thoracic and Tuberculosis Association, 1976; British Thoracic Society, 1984). Therefore, significant effort has been devoted to determining the mechanism of action of POA, a topic that has long been debated (Zhang and Mitchison, 2003; Lamont et al., 2020), and multiple molecular targets have been proposed (Shi et al., 2014; 2011; Kim et al., 2014; Peterson et al., 2015; Njire et al., 2017; Gopal et al., 2020).

**SCHEME 1:**
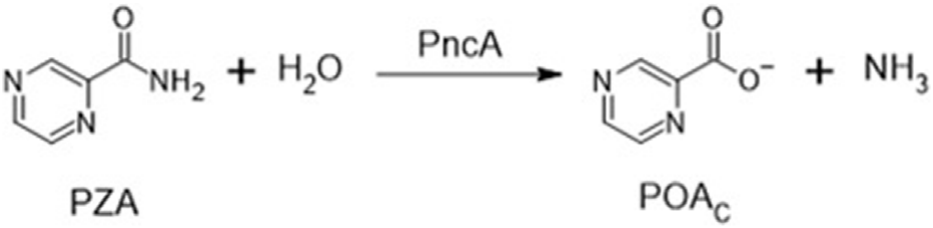
Deamidation of pyrazinamide by the endogenous *Mycobacterium tuberculosis* nicotinamide deamidase PncA.

Intriguingly, the activity of PZA *in vitro* is pH-dependent (Peterson et al., 2015; Salfinger and Heifets, 1988), and two decades ago, it was suggested that the activity of PZA could be predicted using the Henderson–Hasselbalch equation [HHE, (Zhang and Mitchison, 2003)], a hypothesis which was confirmed recently (Fontes et al., 2020). PZA is deaminated in the bacterial cytoplasm by a nicotinamidase (PncA) forming deprotonated pyrazinoate (POA_C_) to exhibit antibacterial activity (Scheme 1) (Konno et al., 1967; Scorpio and Zhang, 1996; Boshoff and Mizrahi, 2000; French et al., 2010). However, of the proposed molecular drug targets, all are cytoplasmic enzymes or activities (Shi et al., 2014; 2011; Kim et al., 2014; Peterson et al., 2015; Njire et al., 2017; Gopal et al., 2020), and none provides an adequate explanation for the pH dependence of PZA activity as *Mycobacterium tuberculosis* maintains cytoplasmic pH homeostasis over an environmental pH range of 5.5–7.3 (Fontes et al., 2020). Recent evidence demonstrated that the growth-inhibitory effect of pyrazinoic acid (POA_N_) on *M. tuberculosis* was due to the acid–base equilibrium and indicated that the POA_C_ formed inside the cell by the activity of PncA must be exposed to the exterior milieu in order to demonstrate a pH effect. However, the chemical nature of a weak acid such as pyrazinoic acid (POA_N_) is seldom considered when the mode of action of this drug is investigated. Here, the effects of exposure of *M. tuberculosis* to structural analogs of POA_N_ and various anti-TB drugs used in the clinic were investigated with the goal of developing a better understanding of the mechanism of action of POA_N_.

**SCHEME 2:**
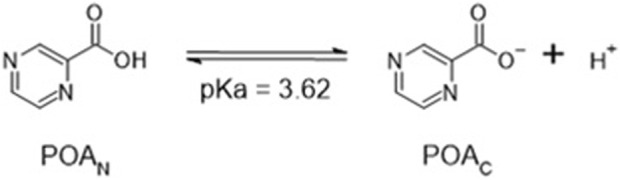
Acid–base equilibrium of pyrazinoic acid.

In solution, the charged, deprotonated POA_C_ resulting from the deamidation of PZA exists in equilibrium with the neutral, protonated POA_N_ (Scheme 2; Supplementary Figure S1), and the total pyrazinoic acid (POA_T_) concentration in solution consists of the sum of the concentrations of the neutral and charged forms (POA_N_ + POA_C_, respectively). The pH-dependent growth inhibitory effect of PZA in *M. tuberculosis in vitro* depends on the differential concentration of POA_N_ in the bacillary cytoplasm and the environment (Fontes et al., 2020). This observation resulted in the hypothesis that POA_N_ may act as a protonophore, uncoupling oxidative phosphorylation and leading to a cascade of secondary effects that cause growth arrest and may explain the synergism PZA exhibits with other anti-tubercular drugs.

Chemiosmotic theory postulates that the establishment of a proton electrochemical gradient across the membrane is generated through oxidative processes and then used to generate ATP (oxidative phosphorylation) (Mitchell, 1966). Substrate oxidation in the electron transport chain drives the generation of an electrochemical potential, designated as proton motive force (PMF), with two associated components: the electric potential (ΔΨ), stemming from all charged species on both sides of the membrane, and the proton concentration gradient (ΔpH) across the plasma membrane. The dissipation of one or both of these components can lead to the disruption of PMF, although compensatory mechanisms have been reported (Bakker and Mangerich, 1981; Booth, 1985).

**FIGURE 1:**
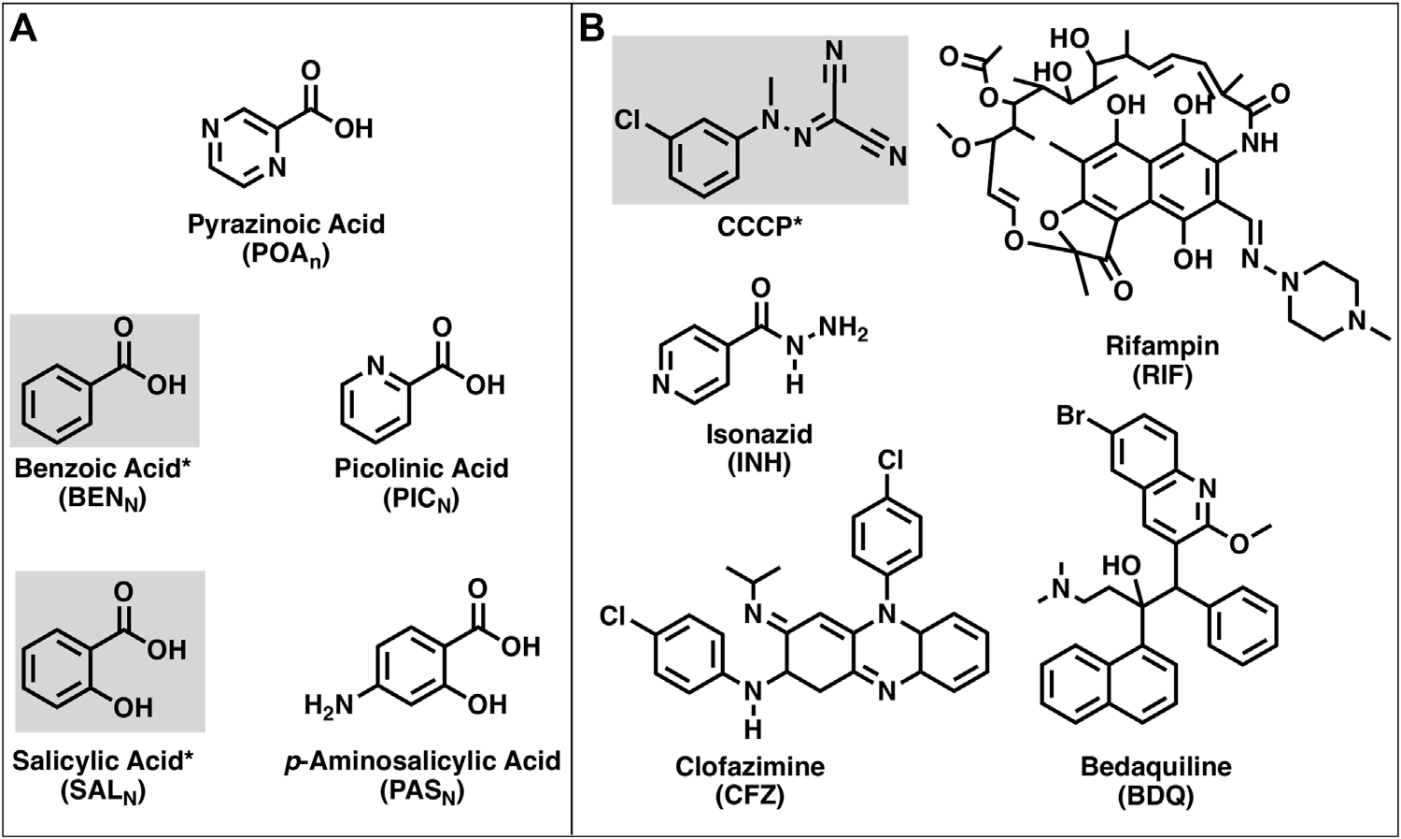
Structures of the compounds used in this work. **(A)** Structures of pyrazinoic acid and analogs. **(B)** Structures of CCCP and various anti-TB drugs. Compounds highlighted and indicated by an asterisk are known protonophores.

Uncouplers of oxidative phosphorylation are compounds able to, through various mechanisms, decouple substrate oxidation from the phosphorylation of ADP to form ATP (Hanstein, 1976). Protonophores are compounds that can dissipate both ΔΨ and ΔpH by shuttling protons across the membrane uncoupling oxidative phosphorylation. Carbonyl cyanide *m*-chlorophenyl hydrazone (Figure 1) is a well-characterized example of such a compound. In solution, the neutral, protonated *N*-(3-chlorophenyl)carbonohydrazonoyl dicyanide (CCCP_N_) exists in equilibrium with its charged, deprotonated form, 1-(3-chlorophenyl)-2-(dicyanomethylene) hydrazine-1-ide (CCCP_C_), and the total carbonyl cyanide *m*-chlorophenyl hydrazone (CCCP_T_) is the sum of the concentrations of each species. The equilibrium between CCCP_N_ and CCCP_C_ drives the activity as a protonophore, in an acidic environment outside the cell, a fraction of CCCP_T_ exists as CCCP_N_. The membrane permeability to CCCP_N_ is greater than that of CCCP_C_ (LeBlanc, 1971), which results in CCCP_N_ being more likely to cross the membrane than CCCP_C_. Upon exposure to the cytoplasmic environment, the higher pH alters the acid–base equilibrium between CCCP_N_ and CCCP_C_ as described by the HHE, releasing protons in the cytoplasm. Therefore, CCCP_N_ disrupts ΔpH if the flux into the cells is greater than the flux of protons generated by the cell in the opposite direction (Mitchell, 1966). Furthermore, since the flux of CCCP_C_ occurs without the use of a counterion, the uncoupling event caused by CCCP_T_ is electrogenic, leading to the dissipation of ΔΨ.

The activity of protonophores, in general, is pH-dependent as the membrane penetration of the protonated form of the compound is dependent on concentration, which, in turn, depends on the total protonophore concentration and the pH of the solution as described by the HHE. Protonophores have simple structural requirements for activity that include a protonatable (weak acid) group for activity and a hydrophobic moiety, often aromatic, that increases membrane solubility (Hanstein, 1976; Heytler, 1979). Many small, hydrophobic weak acids have been shown to be protonophores (Dilger and McLaughlin, 1979; McLaughlin and Dilger, 1980; Gutknecht, 1990; Norman et al., 2004; Peters et al., 2016). These include compounds structurally similar to POA such as benzoic acid and salicylic acid (Figure 1). Other weak acids that are structurally similar to POA include picolinic acid and the anti-TB drug *p*-aminosalicylic acid (Lehmann, 1946a; 1946b). The availability of these compounds and anti-mycobacterial drugs (isoniazid, clofazimine, bedaquiline, and rifampin) that have known mechanisms of action, as well as protonatable groups (Figure 1) that could, in theory, provide protonophoric activity, has enabled the present studies into the potential mechanism of action of PZA.

## Results

### *Mycobacterium tuberculosis* H37Ra pH-dependent growth inhibition

**FIGURE 2:**
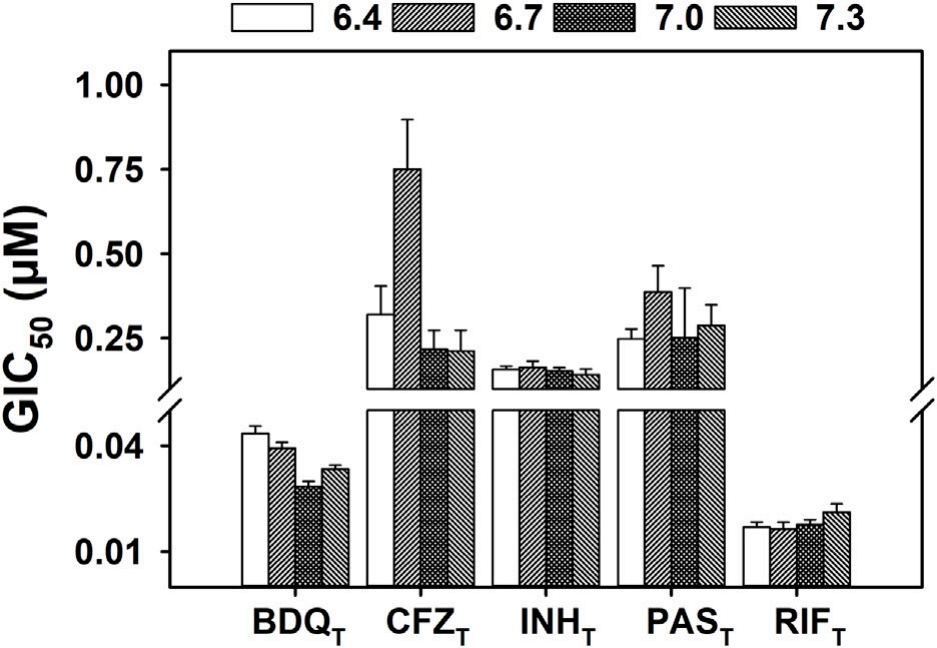
Effect of pH on the growth inhibition of *M. tuberculosis* by known anti-tubercular drugs. *M. tuberculosis* H37Ra were incubated at 37°C in supplemented 7H9 broth with a range of concentrations of the specified antibiotic. The OD_600_ was measured every 24 h for 5 days, with growth rates calculated by determining the slope at each time point. The GIC_50_ (concentration of compound that inhibits 50% of the growth rate) values were calculated with the growth rates of day 5, from four independent biological replicates. The GIC_50_ values extracted from the E_max_ model regression are presented at each external pH tested (6.4–7.3). The error bars of each bar correspond to the standard deviation calculated during the regression. Linear regression analysis of the data can be found in Supplementary Material.

The pH-dependent activity of POA_T_ *in vitro* has been established in multiple reports (Salfinger and Heifets, 1988; Zhang et al., 2002; Peterson et al., 2015; Fontes et al., 2020). However, the effect of pH on the activity of other anti-tubercular drugs is rarely considered, despite the pathogenesis models for *M. tuberculosis* suggesting exposure to a range of pH environments in the host (Effros and Chinard, 1969; Nielson et al., 1981; Nyberg K et al., 1992; Masuda et al., 2015; Irwin et al., 2016; Lanoix et al., 2016). Figure 2 shows the concentrations of the anti-TB drugs *para*-aminosalicylic acid (PAS_T_), bedaquiline (BDQ_T_), clofazimine (CFZ_T_), isoniazid (INH_T_), and rifampin (RIF_T_) responsible for 50% inhibition of *M. tuberculosis* H37Ra growth (GIC_50_) over a small range of environmental pH values (6.4–7.3) corresponding to those likely to be encountered in a host organism. All of the anti-mycobacterial drugs shown in Figure 2 have different molecular targets, and, with the exception of BDQ_T_ (Hards et al., 2018), there is no evidence reported in the literature of ionophoric activity by any of the drugs. Linear regression analysis (Supplementary Table S1) of the data shown in Figure 2 demonstrates that the compounds tested exhibit little or no pH-dependent activity against *M. tuberculosis*, over the pH range tested.

**FIGURE 3:**
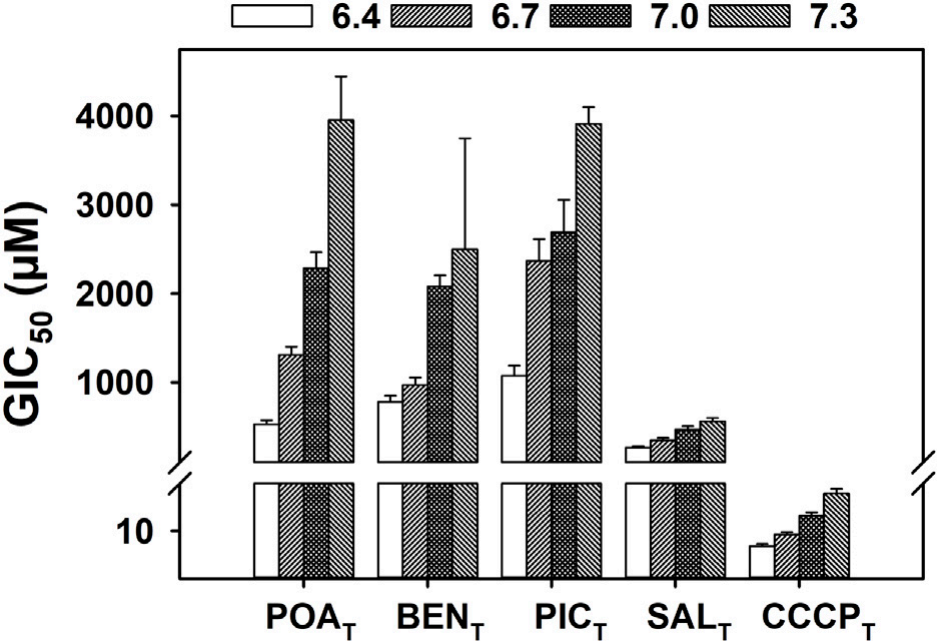
Effect of pH on the growth inhibition of *M. tuberculosis* by POA_T_, structural analogs of POA_T_, and CCCP_T_. Mycobacterial cultures were grown in supplemented 7H9 broth at 37°C and incubated with a range of concentration of the specified compound for 5 days. The OD_600_ was measured every 24 h, and growth rates were determined by calculating the slope of the growth curve at each time point. The GIC_50_ (concentration of compound that inhibits 50% of the growth rate) values shown were determined with the growth rates of day 5 regression of an E_max_ model for each environmental pH value (6.4–7.3), using four biological replicates. The error bars shown correspond to the standard deviation extracted during the regression. Linear regression analysis of the data can be found in Supplementary Material.

Figure 3 shows the GIC_50_ values of POA_T_ and analogs other than PAS_T_ (Figure 2), as well as CCCP_T_ over the pH range of 6.4 and 7.3. POA_T_, SAL_T_, BEN_T_, PIC_T_, and CCCP_T_ all exhibit a linear pH-dependent increase in GIC_50_ as the pH increases with R^2^ values of > 0.9 (Supplementary Table S1). Additionally, CCCP_T_ exhibits the highest efficacy in growth inhibition of all the compounds shown in Figure 3, with SAL_T_ showing the lowest pH-dependent GIC_50_ values among the POA_T_ analogs (including POA_T_). All the compounds shown in Figure 3 also exhibited higher GIC_50_ values than the results for the compounds shown in Figure 2, including the POA_T_ analog PAS_T_.

### Modeling pH-dependent activity in *Mycobacterium tuberculosis* H37Ra

**FIGURE 4:**
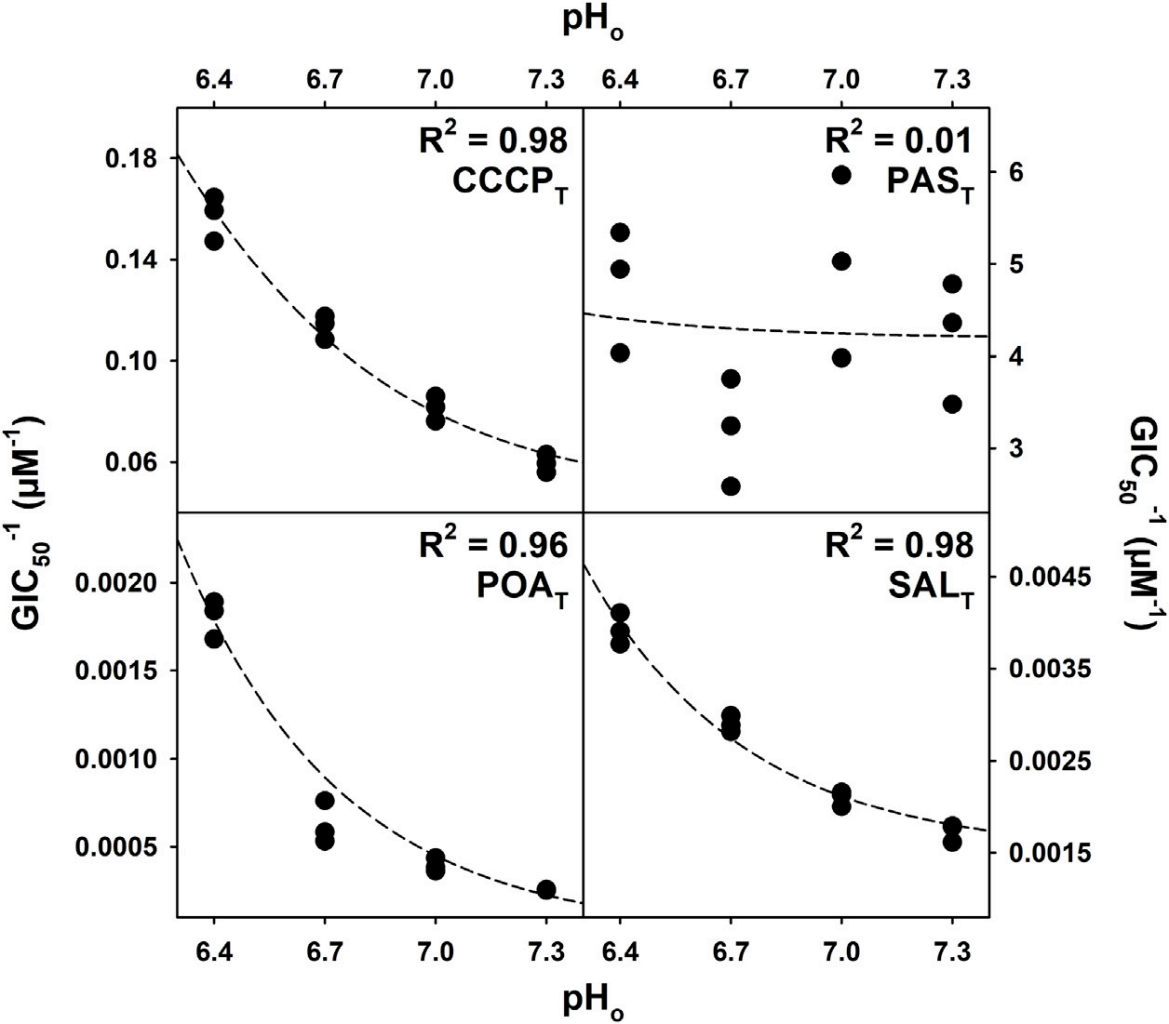
Regression of the QSAR model for CCCP_T_, PAS_T_, POA_T_, and SAL_T_. The GIC_50_ (concentration of compound that inhibits 50% of the growth rate) values (averages of three biological replicates) obtained during the drug susceptibility assay were fit to the model described by Eq. 1. The R^2^ values were calculated based on the residuals of the regression, and the curves generated with the coefficients were determined during the regression.

Quantitative structure–activity relationship (QSAR) models have previously been used to determine the activity of drugs or poisons with pH-dependent properties. The model presented in Eq. 1 (*Materials and methods*) was adapted from the work of Könemann and Musch (1981) and derived from acid–base equilibrium chemistry (Supplementary Equations S1–14; Supplementary Figure S1, S2). The GIC_50_ values obtained during the drug susceptibility assays were fit to the model (Eq. 1), and the coefficients T_N_ and T_C_ were extracted. T_N_ is the inverse of the concentration of the protonated (neutral) form of the compound tested responsible for 50% of the growth inhibition, and T_C_ is the inverse of the concentration of the deprotonated form of the compound tested responsible for 50% of the growth inhibition caused by the anionic species. Thus, both T_N_ and T_C_ are expressed as µM^−1^. The coefficients T_N_ and T_C_ and the coefficient of determination (R^2^) of the fit to the model for each of the compounds used are presented in Table 1. As expected, the compounds that showed no pH-dependent activity did not fit the QSAR model; however, the compounds with clear pH-dependent activities fit the model with R^2^ values greater than 0.9. The modeled behavior of CCCP_T_-, POA_T_-, SAL_T_-, BEN_T_-, and PIC_T_-treated bacterial growth is similar, suggesting similar pH-dependent responses. Figure 4 shows graphic representations of the fits, including the experimentally measured GIC_50_ points, of CCCP_T_, PAS_T_, POA_T_, and SAL_T_, as a function of the environmental pH used for each GIC_50_ determination. Moreover, all of the compounds with high R^2^ scores have values of T_N_ that are much greater than T_C_ (Table 1), indicating a higher efficacy of the protonated form of the compounds and/or a mechanistic requirement for the formation of the protonated form (the latter being reported to be true for the protonophoric activity of both CCCP_T_ and SAL_T_) (Kasianowicz et al., 1984; Norman et al., 2004).

**TABLE 1:** QSAR model coefficients.

Compound	pK_a_ ^a^	T_N_ (µM^−1^)	T_C_ (µM^−1^)	R^2^
No pH-dependent activity				
BDQ_T_	8.91	24 ± 2	360 ± 160	0.30
CFZ_T_	6.63	0 ± 2	6 ± 1	0.30
INH_T_	3.35	0 ± 840	6.3 ± 0.4	0.01
PAS_T_	3.68	100 ± 460	4.0 ± 0.5	0.01
RIF_T_	6.55	50 ± 7	60 ± 4	0.00
pH-dependent activity				
POA_T_	3.62	1.10 ± 0.09	0 ± 0.0001	0.96
BEN_T_	4.08	0.20 ± 0.02	0.0003 ± 0.0001	0.94
PIC_T_	5.52	0.007 ± 0.001	0.0001 ± 0.0001	0.91
SAL_T_	2.79	10 ± 0.5	0.0015 ± 0.0001	0.98
CCCP_T_	5.81	0.60 ± 0.02	0.050 ± 0.003	0.98

^a^ pK_a_ values were obtained through Chemicalize (www.chemicalize.com). For compounds with multiple pK_a_ values, the pK_a_ closest to neutral pH was used for calculations.

### Cytoplasmic acidification by structural analogs of pyrazinoic acid in *Mycobacterium tuberculosis* H37Ra

**FIGURE 5:**
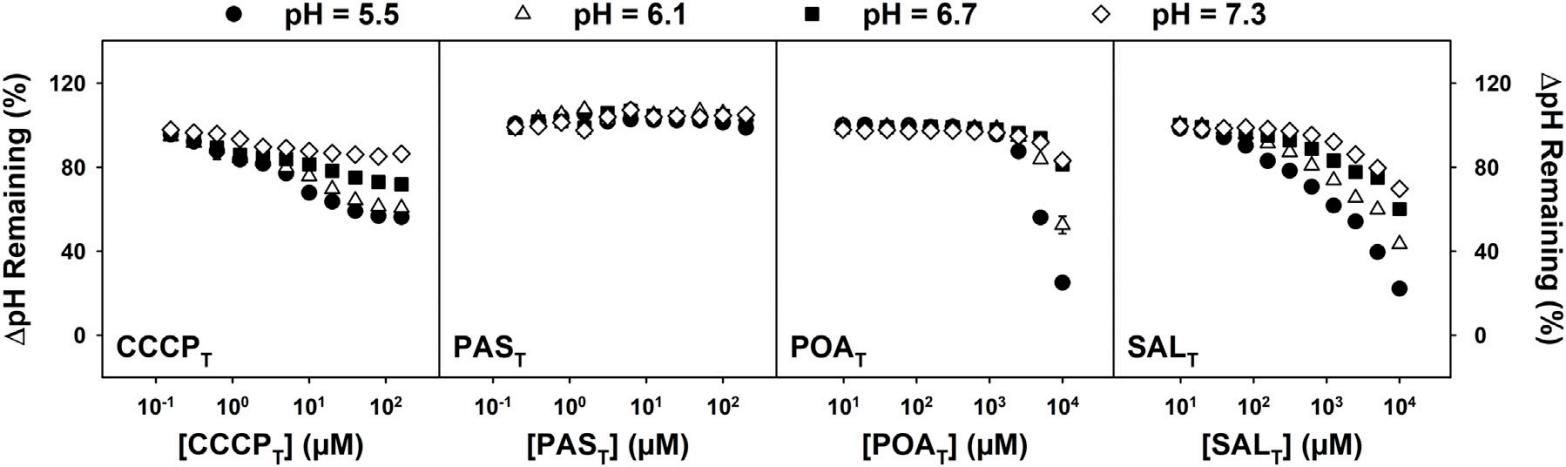
Effects of CCCP_T_, PAS_T_, POA_T_, and SAL_T_ on ΔpH over a range of external pH values. *M. tuberculosis* H37Ra cells were incubated in MMA buffer containing 0.5 µM BCECF-AM at the indicated pH and concentration of the desired analog for 30 min. Fluorescence was measured at 37°C with excitation at 440 and 485 nm and emission recorded at 540 nm The percentage of ΔpH remaining was estimated with the BCECF’s 485/440 ratio after 30 min of incubation with analog divided by the estimated ΔpH (using pH 7.4 as the cytoplasmic pH); the untreated (DMSO control) was considered 100%. Values shown are the averages of four biological replicates +/− standard deviation for each concentration of compound (in most cases, the error bars are buried within the symbols used).

Previous work described POA_T_-induced acidification of the cytoplasm of *M. tuberculosis* H37Ra and suggested the uncoupling of proton motive force by POA_N_ (Fontes et al., 2020). Similarly, PZA has been shown to acidify the mycobacterial cytoplasm (Peterson et al., 2015; Santucci et al., 2022). However, it was not clear that cytoplasmic acidification was a specific effect of POA_T_ or of anti-TB drugs and protonophores in general. Figure 5 shows the change in the pH gradient between the cytoplasm and the exterior environment (ΔpH) across a range of environmental pH values when the mycobacteria cultures were exposed to CCCP_T_, PAS_T_, POA_T_, or SAL_T_. CCCP_T_, POA_T_, and SAL_T_ cause cytoplasmic acidification in a concentration-dependent manner over the range of effective growth inhibition of each compound. In each case, higher efficiency of acidification was observed at the lowest environmental pH tested. However, PAS_T_, which does not show pH-dependent growth inhibition activity, does not acidify the cytoplasm.

## Discussion

### pH-dependent drug susceptibility in *Mycobacterium tuberculosis* H37Ra

The effect of environmental pH on the activity of non-PZA anti-mycobacterial drugs is seldom studied. The drugs used in this work were chosen as they inhibit a range of molecular targets in *M. tuberculosis*. Both BDQ_T_ and CFZ_T_ target energy production, albeit with different modes of action (Yano et al., 2010; Hards et al., 2015), while INH_T_ has been linked to multiple targets, including cell wall biosynthesis inhibition, nucleic acid synthesis inhibition, and reactive oxygen and nitrogen species generation (Gangadharam et al., 1963; Winder et al., 1970; Takayama et al., 1972; Shoeb et al., 1985; Wengenack and Rusnak, 2001; Sipe et al., 2004; Timmins et al., 2004). PAS_T_ is reported to inhibit the folate pathway after endogenous conversion into the active form (Chakraborty et al., 2013; Zheng et al., 2013), and RIF_T_ is a well-described, broad-spectrum inhibitor of DNA-dependent RNA polymerase (Lancini et al., 1969). As shown in Figure 2, none of the anti-mycobacterial drugs mentioned above exhibit pH-dependent increase in the activity as the pH was lowered within the range of environmental pH values tested. The observations of Bartek et al. (2016) support the results shown here for RIF_T_, but for the remaining antibiotics mentioned above, no previous reports on their activity at different environmental pH values were available.

The weak acids, POA_T_ and CCCP_T_, and the structural analogs of POA_T_, other than PAS_T_, all exhibited pH-dependent growth inhibition (Figure 3), in contrast to the results shown for the anti-TB drugs in Figure 2. That is, POA_T_, BEN_T_, SAL_T_, PIC_T_, and CCCP_T_ all demonstrate similar patterns of increasing GIC_50_ as the culture pH is increased over a range of 6.4–7.3. The results imply that these five weak acids likely act via a similar mechanism. Of these compounds, BEN_T_, SAL_T_, and the structurally unrelated CCCP_T_ are well-known and -characterized protonophores (Levitan and Barker, 1972; Neumcke and Bamberg, 1975; McLaughlin and Dilger, 1980; Kasianowicz et al., 1984; Gutknecht, 1990; Norman et al., 2004; Naven et al., 2013; Peters et al., 2016), suggesting that POA_N_ also acts as a protonophore, as previously hypothesized (Fontes et al., 2020).

### Modeling pH-dependent activity in *Mycobacterium tuberculosis* H37Ra

The QSAR model represented by Eq. 1 was first used as a model for the pH-dependent toxicity of phenols in fish (Tabata, 1962; Könemann and Musch, 1981). Phenols, such as 2,4-dinitrophenol, are also known protonophores and uncouple oxidative phosphorylation (Mitchell, 1966; Naven et al., 2013; Childress et al., 2018; Kotova and Antonenko, 2022). The theoretical framework of the model accounts for the independent toxicity of the protonated and deprotonated forms of uncouplers, expressed by the coefficients T_N_ and T_C_, respectively.

Equation 1 was used to determine how well the growth inhibition data for *M. tuberculosis* H37Ra suspensions exposed to anti-tubercular drugs, known protonophores, and weak acids fit the model of protonophoric activity at pH values of 6.4, 6.7, 7.0, and 7.3. The coefficients extracted, when the GIC_50_ values obtained at different environmental pH values for each of the compounds used were fit to the model, are shown in Table 1. The growth inhibition data for the compounds exhibiting pH-dependent activity (Figure 3) fit the model well with R^2^ values greater than 0.9 in all cases. However, the data for those compounds showing no dependence on the pH of the culture medium (Figure 2) do not fit the model. The results of the non-linear regression analysis of growth inhibition data for CCCP_T_, PAS_T_, POA_T_, and SAL_T_ fit to the model, as represented graphically in Figure 4.

The coefficients presented in Table 1 are consistent with the observation that POA_N_ is the active form of PZA (Fontes et al., 2020) as the coefficient T_N_ is orders of magnitude greater than T_C_. The negligible value of T_C_ (relative to T_N_) indicates that the contribution of the deprotonated POA_C_ to growth inhibition is also negligible, and the observable effect relies solely on the concentration of the protonated form (POA_N_). Similarly, the structurally unrelated CCCP_T_ and POA_T_ analogs, other than PAS_T_, follow the same pattern, further supporting the hypothesis of a common mechanism of action.

The concentration of POA_N_ responsible for 50% of the growth inhibition (1/T_N_) corresponds to ∼0.9 µM, a value consistent with the concentration of POA_N_ responsible for 50% of mycobacterial growth inhibition reported previously (Fontes et al., 2020). The corresponding concentrations for SAL_N_, BEN_N_, PIC_N_, and CCCP_N_ are 0.1, 5.0, 140, and 1.7 µM, respectively. Thus, POA_N_ is twice as effective at inhibiting *M. tuberculosis* growth as CCCP_N_. Moreover, the inverse T_N_ concentration for SAL_N_ is ∼0.1 µM, which would make SAL_N_ the best compound at inhibiting mycobacterial growth via a protonophoric mechanism. However, the low pK_a_ values of POA_T_ and SAL_T_, 3.62 and 2.79, respectively, dictate that to achieve the concentrations of POA_N_ and SAL_N_ required to inhibit 50% of growth in environments near pH neutrality, the concentrations of POA_T_ and SAL_T_ must be orders of magnitude higher. In addition, given the higher pK_a_ of CCCP_T_ (5.81), concentrations of total compound are closer to the CCCP_N_ concentration needed to achieve a similar effect. As a result, the concentration of CCCP_T_ required to inhibit 50% of bacterial growth is orders of magnitude lower than the concentrations of POA_T_ and SAL_T_. However, it is essential to remember that the activity of a protonophore is not solely dependent on pKa. If one plots 1/T_N_ vs. pKa for the pH-dependent compounds used in this work and conducts a linear regression analysis, the resulting R^2^ value is less than 0.3. This is likely due, at least in part, to the observation that the rate of PMF dissipation is dependent on the rate of diffusion of the anionic form of the protonophore across the membrane rather than the rate of diffusion of the neutral form in the other direction, which is much greater (McLaughlin and Dilger, 1980). Nonetheless, the results strongly suggest that POA_N_ acts as a protonophore, given all the similarities described with the known protonophores CCCP_N_, BEN_N_, and SAL_N_ and the fact that it is unlikely that these compounds would all bind to the same molecular target with similar affinity (resulting GIC_50_ values in the 0.1–5 µM range).

Figure 4 also shows the poor fit obtained for PAS_T_ growth inhibition data, which reflects the lack of pH dependence presented in Figure 2. PAS_T_ is a weak acid analog of POA_T_, as are SAL_T_, BEN_T_ and PIC_T_, which might be expected to have a similar pH behavior and mechanism of action. Nevertheless, the drug susceptibility assays and the QSAR model used here indicate otherwise. This is in agreement with the report that PAS_T_ inhibits the folate pathway in *M. tuberculosis* after conversion to hydroxyl-dihydropteroate with an MIC_50_ of 0.4 µM. (Zheng et al., 2013). The existence of an endogenous cytosolic molecular target causes bacterial growth inhibition of *M. tuberculosis* at concentrations orders of magnitude lower than those where a protonophoric activity would be observable given the PAS_T_ pKa of 3.25. Thus, the QSAR model is sensitive to the presence of high-affinity, cytosolic molecular targets.

Similarly, the drug susceptibility assays and the QSAR model used here did not indicate that BDQ_T_ had pH-dependent or ionophoric activity, observations which are supported by Haagsma et al. (2011), even though BDQ_T_ was reported to act as an H^+^/K^+^ antiporter ionophore in *E. coli* membrane vesicles (Hards et al., 2018). In *M. tuberculosis*, BDQ_T_ inhibits the F_1_F_0_-ATP synthase, binding to the a-c subunit of the F_0_ complex of the ATP synthase and leading to depletion of ATP (Hards et al., 2015). However, *E. coli* ATP synthase lacks the BDQ_T_-binding site (Preiss et al., 2015), and the concentrations needed to disrupt PMF in *E. coli* were 30–300-fold higher than the BDQ_T_ MIC in *M. tuberculosis*. (Hards et al., 2018).

### Cytoplasmic acidification by structural analogs of pyrazinoic acid in *Mycobacterium tuberculosis* H37Ra

Consistent with a lack of pH dependence and the QSAR analysis, PAS_T_ causes no change in the mycobacterial transmembrane ΔpH over the range of concentrations that generates PAS_T_-induced growth inhibition. However, Figure 5 shows that cytosolic acidification is apparent when *M. tuberculosis* H37Ra cultures are treated with POA_T_, SAL_T_, and CCCP_T_. This is consistent with the previous observations that POA_T_ causes rapid cytosolic acidification and dissipation of ΔΨ in *M. tuberculosis*. The cytosolic acidification caused by CCCP_T_ is supported by observations described in the work of Peterson et al. (2015) and Fontes et al. (2020). Zhang et al. (2003) showed similar GIC_50_ values for BEN_T_ and SAL_T_ to the values presented here, but they observed no change in the internal pH caused by the two compounds (Zhang et al., 2003). However, these researchers did demonstrate that BEN_T_ and SAL_T_ induced ΔΨ disruption in *M. tuberculosis* H37Ra.

The curves presented in Figure 5 suggest that the mechanism of acidification of the cytoplasm by CCCP_T_ is potentially distinct from that of POA_T_ and SAL_T_. This may be a reflection of the different protonophoric mechanisms reported for CCCP_T_ and SAL_T_ (Neumcke and Bamberg, 1975; McLaughlin and Dilger, 1980; Kasianowicz et al., 1984; Gutknecht, 1990; Norman et al., 2004). The mechanism of CCCP_T_ action (termed A^−^) involves the permeation of the protonated CCCP_N_ through the membrane, the release of the proton upon arrival in the cytoplasm, and subsequent diffusion of the deprotonated CCCP_C_ through the membrane to the extracellular medium, where CCCP_C_ can be protonated regenerating CCCP_N_, and the cycle may begin again (Neumcke and Bamberg, 1975; McLaughlin and Dilger, 1980; Kasianowicz et al., 1984). The uncoupling mechanism of SAL_T_ has been shown to be distinct from the A^−^ mechanism (McLaughlin and Dilger, 1980; Gutknecht, 1990; Norman et al., 2004). The SAL_T_ mechanism, termed AHA^−^ (or HA_2_^-^), involves the protonated form of the uncoupler permeating the membrane and releasing a proton on the cytoplasmic side of the membrane, just as described with the A^−^ mechanism. However, the AHA^−^ mechanism requires the deprotonated form to establish a heterodimer with a protonated form of itself, forming an anionic dimer (in the case of SAL_T_, it requires the SAL_C_ to form a complex with a molecule of SAL_N_) (Neumcke and Bamberg, 1975; McLaughlin and Dilger, 1980). The dimer is, then, able to permeate the membrane, and the deprotonated form of the uncoupler can reach the extracellular medium, where it is available to be protonated again and to initiate a new cycle. Both mechanisms acidify the cytoplasm and are electrogenic, altering both ΔΨ and ΔpH and disrupting PMF (Kasianowicz et al., 1984; Gutknecht, 1992; Norman et al., 2004). In both mechanisms, the concentration of each weak acid must be such that the effective proton conductance is greater than the sum of all other membrane conductance; in addition, the buffers used, solubility in and diffusion through the unstirred water layers adjacent to the membrane, the electrostatic potentials at the membrane–solution interface, the thickness of the bilayer, and the dielectric constant of the membrane all modify the protonophore activity of each weak acid (McLaughlin and Dilger, 1980). While SAL_T_ has many other reported mechanisms of action (Klessig et al., 2016), the evidence presented here indicates that SAL_T_ behaves solely as a protonophore in *M. tuberculosis*, and the data suggest that the protonophoric activity of POA_T_ may have a similar AHA^−^ mechanism.

Previous reports have shown the susceptibility of *M. tuberculosis* H37Ra and *M. smegmatis* to growth inhibition by SAL_T_ and BEN_T_, among other weak acids (Zhang et al., 2003). Furthermore, the activity of SAL_T_ and BEN_T_ was shown to exhibit sterilizing activity against *M. tuberculosis* H37Ra (Wade and Zhang, 2006). However, the literature on the impact of SAL_T_ or aspirin, the prodrug form of SAL_T_ used therapeutically, in TB infections is scarce and somewhat controversial (Byrne et al., 2007a; Byrne et al., 2007b; Eisen et al., 2013; Vilaplana et al., 2013). While PZA exhibits activity *in vivo*, the integration of the antibiotic in the standard regimen for TB infections only occurred after the discovery of the synergism between PZA and RIF_T_ (British Thoracic and Tuberculosis Association, 1976; British Thoracic Society, 1984). Aspirin and SAL_T_, however, were shown to antagonize the activity of multiple anti-tubercular drugs (Schaller et al., 2002). Still, these studies were conducted in neutral pH environments, where SAL_T_ is less effective, and therefore, the nature of the drug–drug interactions between SAL_T_ and other antibiotics deserves further investigation. Studies conducted on mice seem to agree on the mild antagonism between SAL_T_ and INH_T_, although an increase in bacterial load clearance with a combination of aspirin and INH_T_ was also observed (Byrne et al., 2007a). Moreover, SAL_T_ was shown to synergize with PZA in a murine model (Byrne et al., 2007b), and evidence of improved outcome in patients undergoing treatment with PZA (plus INH_T_ and ethambutol) when aspirin was added to the regimen exists (Horsfall et al., 1979; Schoeman et al., 2011).

In conclusion, the data presented suggest that pyrazinoic acid acts as an uncoupler of oxidative phosphorylation and that the disruption of proton motive force is the primary mechanism of action of pyrazinoic acid rather than the inhibition of a classic enzyme activity.

## Materials and methods

The reagents used in this work were purchased from Sigma-Aldrich, except when noted. Middlebrook 7H9 broth was purchased from Becton Dickinson and albumin from GoldBio. The fluorescent dye used in the cytosolic acidification assay was obtained from Invitrogen. All reagents were used without further purification and were, at least, of reagent grade.

### *Mycobacterium tuberculosis* H37Ra culture methods

*M. tuberculosis* H37Ra cultures were grown as reported elsewhere (Fontes et al., 2020). In brief, frozen glycerol stocks of *M. tuberculosis* H37Ra were thawed and used to inoculate the Middlebrook 7H9 medium, which was supplemented with oleic acid-albumin-dextrose (OAD, 10% v/v), 0.1% v/v tyloxapol, and 0.2% w/v casamino acids (supplemented 7H9 broth). The pH of the medium was adjusted with hydrogen chloride or sodium hydroxide, when needed, and verified in all cases. The culture flasks were incubated at 37°C, under constant agitation. The cells were harvested when an optical density of 600 nm (OD_600_) between 0.6 and 0.8 was observed. Further processing of the cultures was carried out according to the requirements of individual assays.

### pH-dependent growth inhibition of *Mycobacterium tuberculosis* H37Ra

The growth inhibition activity of anti-tubercular drugs and POA_T_ analogs was determined, as described previously (Gruppo et al., 2006). Cells of *M. tuberculosis* H37Ra were grown and harvested, with subsequent centrifugation using a Beckman CS-6R centrifuge at 1,500 × g, and then washed with fresh culture medium (supplemented 7H9 broth). The pH of the culture medium was adjusted as described above to obtain pH values ranging from 6.4 to 7.3 in 0.3 unit intervals. Cells were resuspended in the culture medium at the desired pH and incubated until an OD_600_ between 0.6 and 0.8 was reached, as a way to acclimatize the bacilli to the pH of the medium, as described elsewhere (Fontes et al., 2020). The cells were then harvested, centrifuged as above, and washed with fresh medium at the desired pH value. Aliquots of 198 µL of resuspended cells at an OD_600_ of 0.1 were transferred to 96-well microtiter plates containing one zirconia bead per well (diameter approximately 1 mm) to assist with agitation and aeration. Aliquots of 2 µL of a range of concentrations of drug dissolved in dimethyl sulfoxide (DMSO) or water (for BEN_T_), including an aliquot containing no drug as a blank, were added to the plate, and the OD_600_ was recorded using a BioRad Benchmark Plus plate reader. The OD_600_ was determined over 5 days, with readings every 24 h. Incubation between time points occurred at 37°C with constant rocking of the plates within a sealed plastic bag containing a damp paper towel to maintain humidity and prevent evaporation. Growth rates were determined by the calculation of the slope of the growth curve at each time point. The calculation of the concentration required to inhibit 50% of growth was carried out using a non-linear four-parameter E_max_ regression model.

### Modeling uncoupling activity in *Mycobacterium tuberculosis*

Multiple QSAR models have been proposed to determine the activity of analogs of poisons or drugs. However, the impact of pH in the activity of these compounds is seldom taken into consideration. Based on the work of Tabata (1962), Könemann and Musch (1981) derived a model that accounts for the specific activity of each form of an ionizable compound, which is shown in Eq. 1. The derivation of the model is provided in Supplementary Material.

$$\frac{1}{{\text{GIC}}_{50}}=T_{N}∙\frac{10^{-\text{pH}}}{10^{-\text{pH}}+10^{{-\text{pK}}_{a}}}+T_{C}∙\frac{10^{{-\text{pK}}_{a}}}{10^{-\text{pH}}+10^{{-\text{pK}}_{a}}},$$ (1)

where GIC_50_ corresponds to the concentration (total, independent of the protonation state) of a compound required to inhibit 50% of growth, T_N_ corresponds to the inverse of the concentration of the protonated form of the compound required to cause 50% inhibition of the effect, and T_C_ is the inverse of the concentration of the deprotonated form of the compound required to cause 50% inhibition. The pH term corresponds to the environmental pH at which the GIC_50_ value was obtained, and pK_a_ corresponds to the pK_a_ of the protonated form of the compound.

The GIC_50_ values obtained in the drug susceptibility assay described above were fit to the model described by Eq. 1 with an algorithm created in R. As T_N_ and T_C_ correspond to concentrations and, therefore, have biochemical meaning, the algorithm was designed to restrain the values of the coefficients to only values equal or above zero. The coefficients obtained from the fit are reported in Table 1 with standard errors obtained from the fit. The coefficient of determination was calculated with the residuals of the fit.

### Effect of pyrazinoic acid on the internal pH of *Mycobacterium tuberculosis* H37Ra cells

The assessment of the internal pH of mycobacterial cells by analogs of POA_T_ was performed, as reported previously (Fontes et al., 2020). *M. tuberculosis* H37Ra cultures were grown as described above, and the cells were harvested by centrifugation and washed twice with MMA buffer (a mixed buffer consisting of 25 mM MES, 25 mM MOPS, and 50 mM AMP, prepared as previously communicated) (Fontes et al., 2020). Cells were resuspended in MMA buffer at the desired pH to an OD_600_ of 0.3, and aliquots of 196 µL were transferred into a black-walled 96-well titer plate with one zirconia bead in each well. Background fluorescence was recorded at 37°C for 10 min (in 2 min intervals) on a BioTek Synergy HT (excitation at 440 nm and 485 nm and emission at 540 nm), followed by the addition of 0.5 µM of 2′,7′-bis-(2-carboxyethyl)-5-(and-6)-carboxyfluorescein acetoxymethyl ester (BCECF-AM) to the cells. Fluorescence was recorded for 30 min. Aliquots of 2 µL of a range of concentrations of the desired analog of POA_T_ dissolved in DMSO (with DMSO without drug serving as a blank) were then added to the cultures, and fluorescence was recorded for another 30 min.

## Data Availability

The raw data supporting the conclusion of this article will be made available by the authors, without undue reservation.

## Abbreviations

BDQ, bedaquiline; BEN, benzoic acid; CFZ, clofazimine; CCCP, *N*-(3-chlorophenyl)carbonohydrazonoyl dicyanide; INH, isoniazid; PAS, *p*-aminosalicylic acid; PIC, picolinic acid; POA, pyrazinoic acid; PZA, pyrazinamide; RIF, rifampin; and SAL, salicylic acid. The abbreviation used for the protonated form of each of these compounds is followed by a subscripted N and the deprotonated form a subscripted C; when referring to both forms or when the form present is unclear, a subscript T is used.
