# Supplementary material for: Pyrazinoic acid, the active form of the anti-tuberculosis drug pyrazinamide, and aromatic carboxylic acid analogs are protonophores
Source: Front Mol Biosci. 2024 Feb 13;11:1350699. doi: 10.3389/fmolb.2024.1350699 (PMC10896915; doi:10.3389/fmolb.2024.1350699)
Supplement: Supplementary file 1 [file Table1.DOCX]

**Suppplementary Material**

**Pyrazinoic Acid, Active Form of the Anti-Tuberculosis Drug Pyrazinamide, and Aromatic Carboxylic Acid Analogs are Protonophores**

Fabio L. Fontes,^1,2^ Steven A. Rooker,^2^ Jamie K. Lynn-Barbe,^2^ Michael A. Lyons^2^, Debbie C. Crans,^1,3^ and Dean C. Crick^1,2^

^1^Cell and Molecular Biology Program, Colorado State University, Fort Collins, Colorado 80523, United States

^2^Mycobacteria Research Laboratories, Department of Microbiology, Immunology and Pathology, Colorado State University, Fort Collins, Colorado 80523, United States

^3^Department of Chemistry, Colorado State University, Fort Collins, Colorado 80523, United States

**Derivation of the pH‑dependent activity model.**


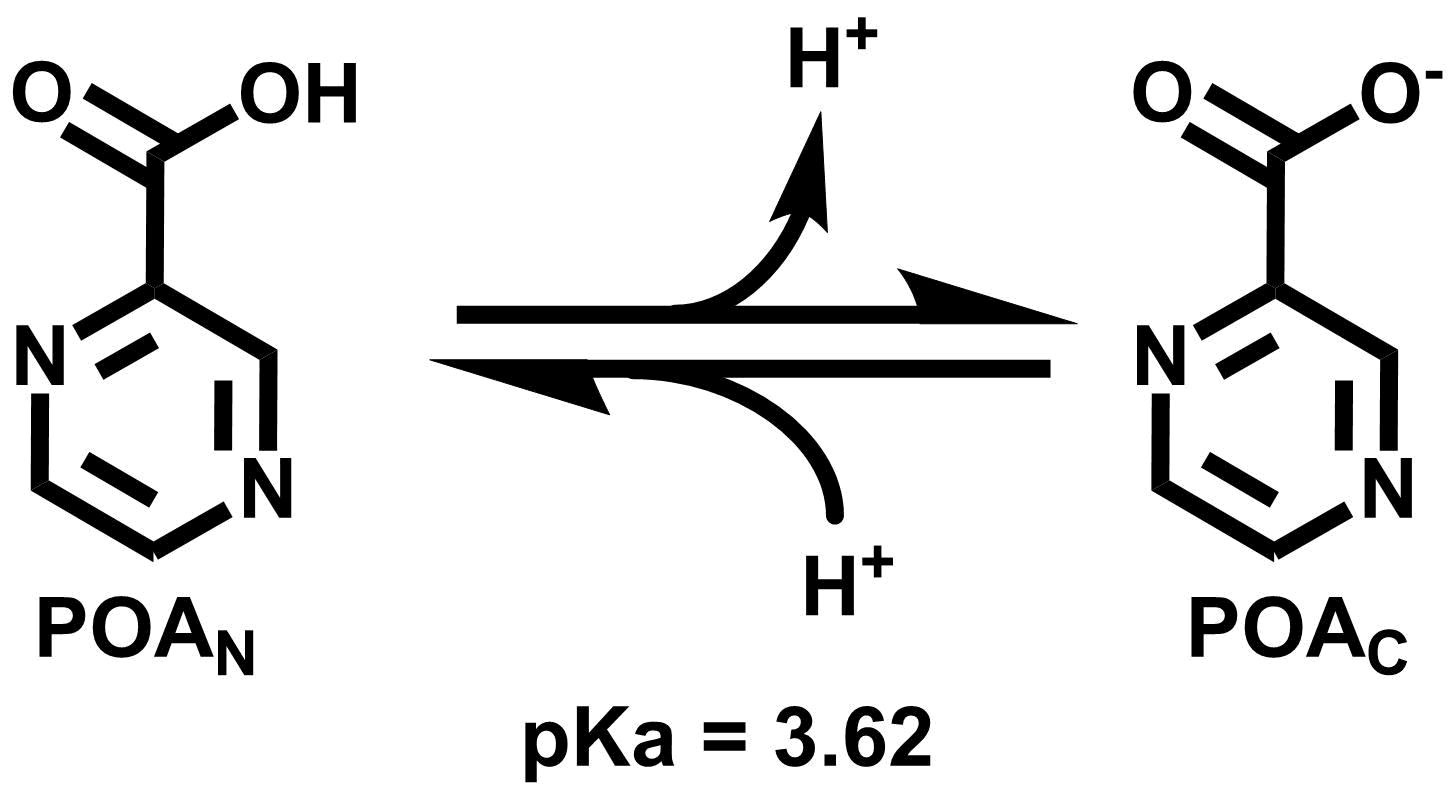


**Figure S1** – Acid‑base equilibrium of pyrazinoic acid (POA_N_) with pyrazinoate (POA_C_). The pK_a_ shown was obtained using Chemicalize (www.chemicalize.com)

Modeling the pH‑dependent activity of relevant drugs or toxins has been the subject of extensive literature. The following model is adapted from Könemann and Musch,^1^ with more detailed explanation and further development and interpretation.

For an ionizable molecule, such as pyrazinoic acid in protonated form (Figure S1, abbreviated as POA_N_), it is theoretically possible for either or both protonated and deprotonated forms of the compound to exhibit an effect. In the example of total POA (POA_T_) both POA_N_ and its deprotonated form, pyrazinoate (POA_C_) could potentially exhibit growth inhibition activity. The model assumes that any observable effect results from the additivity of the effect of both forms of the molecule. Hence, the effect of an ionizable molecule can be expressed as

$\frac{\left[ \mathrm{HA} \right]+\left[ A^{-} \right]}{\mathrm{EC}_{50}}=T_{N}\cdot\left[ \mathrm{HA} \right]+T_{C}\cdot\left[ A^{-} \right]$ Equation S1

where [HA] and [A^-^] stand for the concentrations of the protonated and deprotonated forms of the molecule, respectively. EC_50_ corresponds to the overall concentration of the molecule (or [HA]+[A^-^]) that causes 50% of the observable effect. The specific effect of each form is expressed by T_N_ and T_C_, as the inverse of the concentration responsible for 50% of the effect caused by [HA] (in the case of T_N_) or [A^-^] (in the case of T_C_):

$\frac{1}{\mathrm{EC}_{50}^{N}}=T_{N}$ Equation S2

$\frac{1}{\mathrm{EC}_{50}^{C}}=T_{C}$ Equation S3

with EC_50_^N^ representing the concentration of HA that causes 50% of the effect of HA and EC_50_^C^ representing the concentration of A^-^ responsible for 50% of the effect caused by A^-^.

The ionizable molecule exists in an acid‑base equilibrium in solution. Which, following acid‑base chemistry, can be expressed as

$\frac{\left[ H^{+} \right]\cdot\left[ A^{-} \right]}{\left[ \mathrm{HA} \right]}=K_{a}$ Equation S4

with K_a_ corresponding to the acid equilibrium constant of the reversible reaction

$\mathrm{HA}\rightleftarrows H^{+}+A^{-}$ Equation S5

In which H^+^ represents the proton released in the reaction. With respect to POA_N_ and POA_C_, the equilibrium reaction takes the form of

$\mathrm{POA}_{N}\rightleftarrows H^{+}+\mathrm{POA}_{C}$ Equation S6

When the effect is defined at the concentration EC_50_, Equation S1 can be simplified, as follows:

$\left[ \mathrm{HA} \right]+\left[ A^{-} \right]=\mathrm{EC}_{50}$ Equation S7

allowing for the expression of [HA] and [A^-^] in terms of K_a_, H^+^ and EC_50_, as follows (using Equation S4 and Equation S7):

$\left[ \mathrm{HA} \right]=\frac{\left[ H^{+} \right]}{\left[ H^{+} \right]+K_{a}}\cdot\mathrm{EC}_{50}$ Equation S8

and

$\left[ A^{-} \right]=\frac{K_{a}}{\left[ H^{+} \right]+K_{a}}\cdot\mathrm{EC}_{50}$ Equation S9

Substituting Equation S7, Equation S8 and Equation S9 in Equation S1 yields

$1=T_{N}\cdot\frac{\left[ H^{+} \right]}{\left[ H^{+} \right]+K_{a}}\cdot\mathrm{EC}_{50}+T_{C}\cdot\frac{K_{a}}{\left[ H^{+} \right]+K_{a}}\cdot\mathrm{EC}_{50}$ Equation S10

or

$\frac{1}{\mathrm{EC}_{50}}=T_{N}\cdot\frac{\left[ H^{+} \right]}{\left[ H^{+} \right]+K_{a}}+T_{C}\cdot\frac{K_{a}}{\left[ H^{+} \right]+K_{a}}$ Equation S11

While [H^+^] and K_a_ can be easily calculated, it is commonly to express both in terms of pH and pK_a_, respectively, for commodity of smaller numbers. The relationship between pH and [H^+^] follows

$\left[ H^{+} \right]={10}^{-pH}$ Equation S12

While the relationship between pK_a_ and K_a_ arises, in a similar manner, from

$K_{a}={10}^{{-pK}_{a}}$ Equation S13

and the substitution of these equalities in Equation S11 results in

$\frac{1}{\mathrm{EC}_{50}}=T_{N}\cdot\frac{{10}^{-pH}}{{10}^{-pH}+{10}^{{-pK}_{a}}}+T_{C}\cdot\frac{{10}^{{-pK}_{a}}}{{10}^{-pH}+{10}^{{-pK}_{a}}}$ Equation S14

Equation S14 (or Equation S11, if [H^+^] and K_a_ are used) can be analyzed further if each term of the equation is interpreted as dominant in terms of the corresponding effect of each form of the ionizable molecule. Könemann and Musch make a reference to this differential effect in terms of membrane solubility, suggesting it is common to observe a higher toxicity for the protonated form of phenols.^1^ The dominance of one of the forms can also be interpreted as a specific effect requiring a specific form or an indication of a kinetic limiting step requiring the formation of a specific form of the molecule. Additionally, the dominance of a particular form in the overall effect can reflect a combination of the factors described above. The dominance of a form is expressed by the value of its corresponding term, i.e., if T_N_ has an absolute value much higher than T_C_, the protonated form has a dominant effect compared to the effect resulting from the deprotonated form.


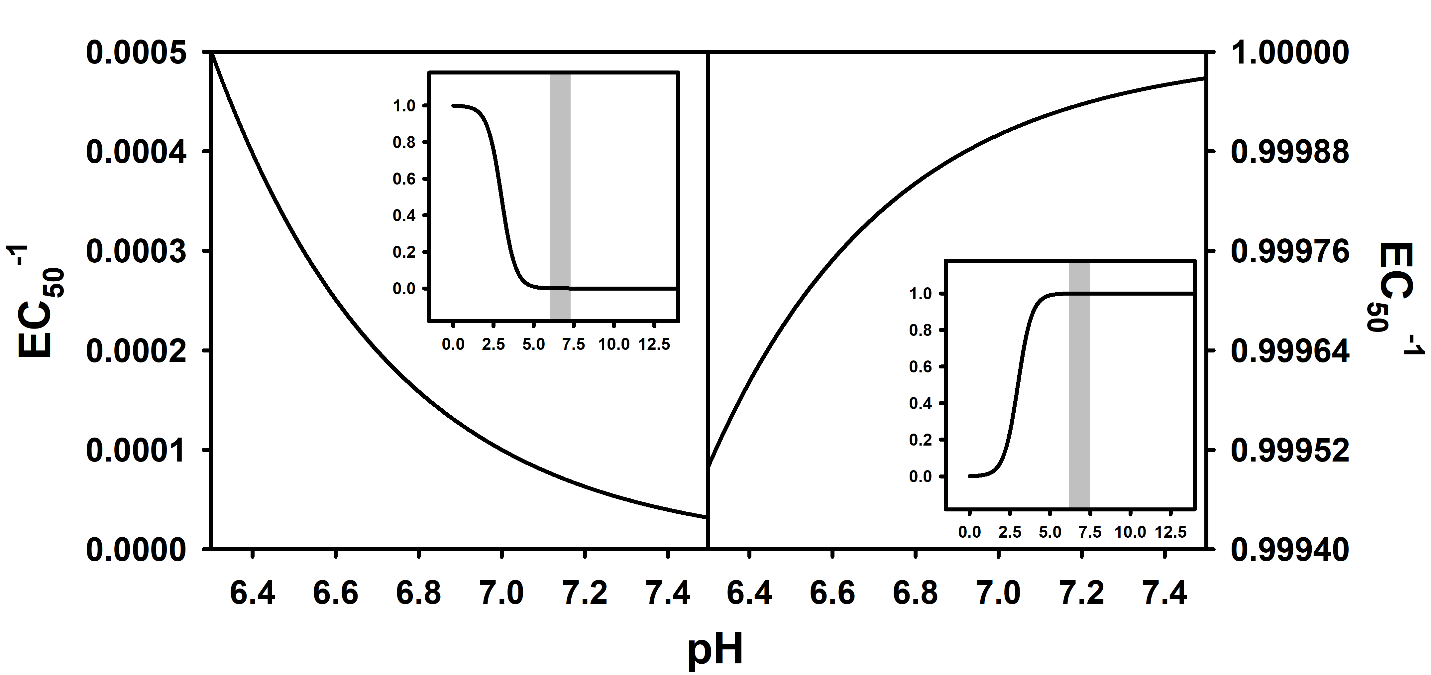


**Figure S2** – Theoretical curves described by Equation S14 where T_N_ = 1 and T_C_ = 0 (left panel) or T_N_ = 0 and T_C_ = 1 (right panel) for a hypothetical compound with a pK_a_ of 3. The insets show the curves over a pH range > 0. The area highlighted in grey is shown in detail in the main panels and corresponds to the pH range likely encountered by M. tuberculosis bacilli in a host organism and studied in this work.

The dominance of a species can also be determined graphically, as seen in Figure S2. The theoretical case of an ionizable molecule with a pK_a_ of 3.0 is shown, with the panel on the left representing dominance of the protonated form (T_N_ = 1 and T_C_ = 0) and the panel on the right dominance of the deprotonated form (T_N_ = 0 and T_C_ = 1) with particular focus on the range of pH values used to determine the growth inhibitory concentrations (GIC_50_) for pyrazinoic acid and the other compounds tested in the present work. As the left panel of Figure S2 shows, when the protonated form has a dominant effect, the inverse of EC_50_ decreases with increasing pH (as is seen in Figure 4 for CCCP, POA_T_ and SAL_T_). Conversely, the right panel of Figure S2 shows the curve of Equation S14 when the deprotonated form dominates the effect and results in an increase in the inverse of EC_50_ values as pH increases. The theoretical case of no dominance (where the numerical value of T_N_ is close to the value of T_C_) would result in a curve with no slope or a small slope. However, these molecules are impossible to distinguish from molecules that have an effect which is not pH‑dependent.

The numerical values of T_N_ and T_C_ presented in Table 2 were determined using non‑linear curve fitting methods, however, Könemann and Musch provide a linearization of Equation S11 that may prove to be useful in cases where non-linear methods are not available or difficult to use.^1^ From Equation S10,

$1=T_{N}\cdot\frac{\left( \left[ H^{+} \right]+K_{a}-K_{a} \right)}{\left[ H^{+} \right]+K_{a}}\cdot\mathrm{EC}_{50}+T_{C}\cdot\frac{K_{a}}{\left[ H^{+} \right]+K_{a}}\cdot\mathrm{EC}_{50}$ Equation S15

as the consecutive addition and subtraction of K_a_ is neutral. Then

$1=T_{N}\cdot\frac{\left[ H^{+} \right]+K_{a}}{\left[ H^{+} \right]+K_{a}}\cdot\mathrm{EC}_{50}-T_{N}\cdot\frac{K_{a}}{\left[ H^{+} \right]+K_{a}}\cdot\mathrm{EC}_{50}+T_{C}\cdot\frac{K_{a}}{\left[ H^{+} \right]+K_{a}}\cdot\mathrm{EC}_{50}$ Equation S16

which can be simplified to

$\frac{1}{\mathrm{EC}_{50}}=T_{N}-\left( T_{N}-T_{C} \right)\cdot\frac{K_{a}}{\left[ H^{+} \right]+K_{a}}$ Equation S17

The resulting Equation S17 generates a linear curve when the inverse of EC_50_ is used in the y‑axis and $\frac{K_{a}}{\left[ H^{+} \right]+K_{a}}$ is used as x‑axis. The resulting curve provides an easy way to determine T_N_, since it corresponds with the y‑intercept and the slope can then be used to determine T_C_.

The derivation presented here used the concentrations that cause 50% of the effect as GIC_50_ values in this work with pyrazinoic acid and the other compounds. However, it is noteworthy that other percentages of effect (such as 90% or 99%) can be used, with the results translating to those percentages of effect across all coefficients (EC, T_N_ and T_C_).

**Reference.**

(1) Könemann, H.; Musch, A. Quantitative structure-activity relationships in fish toxicity studies (Part 2): The influence of pH on the QSAR of chlorophenols. *Toxicology* **1981**, *19* (3), 223–228. https://doi.org/10.1016/0300-483X(81)90131-1.

**Table S1** – Linear Regression analysis of the data shown in Figures 2 and 3.

| Compound | GIC_50_ Change/pH Unit*  (µM/pH unit) | R^2*^ |
| --- | --- | --- |
| No pH-dependent Activity | | |
| BDQ | -0.014 | 0.64 |
| CFZ | -0.29 | 0.19 |
| INH | -0.20 | 0.69 |
| PAS | -0.0057 | 0.0011 |
| RIF | 0.0047 | 0.71 |
| pH-dependent activity | | |
| POA | 3800 | 0.97 |
| BEN | 2100 | 0.93 |
| PIC | 2900 | 0.96 |
| SAL | 340 | 0.99 |
| CCCP | 12 | 0.98 |

* Slopes and R^2^ values were calculated using SigmaPlot 15 (see below). Data points are averages of four replicates.

**BDQ**

**Linear Regression**

**Data source:** Data 1 in Notebook1

Col 2 = 0.129 - (0.0136 * Col 1)

N = 4 Missing Observations = 1

R = 0.801 Rsqr = 0.642 Adj Rsqr = 0.462

Standard Error of Estimate = 0.005

**Coefficient Std. Error t P**

Constant 0.129 0.0494 2.622 0.120

Col 1 -0.0136 0.00720 -1.892 0.199

Analysis of Variance:

**DF SS MS F P**

Regression 1 0.0000835 0.0000835 3.581 0.199

Residual 2 0.0000467 0.0000233

Total 3 0.000130 0.0000434

Normality Test (Shapiro-Wilk) Passed (P = 0.197)

Constant Variance Test (Spearman Rank Correlation): Failed (P = <0.001)

Power of performed test with alpha = 0.050: 0.195

The power of the performed test (0.195) is below the desired power of 0.800.

Less than desired power indicates you are less likely to detect a difference when one actually exists. Negative results should be interpreted cautiously.

**CFZ**

**Linear Regression**

**Data source:** Data 1 in Notebook1

Col 3 = 2.332 - (0.286 * Col 1)

N = 4 Missing Observations = 1

R = 0.434 Rsqr = 0.188 Adj Rsqr = 0.000

Standard Error of Estimate = 0.282

**Coefficient Std. Error t P**

Constant 2.332 2.878 0.810 0.503

Col 1 -0.286 0.420 -0.681 0.566

Analysis of Variance:

**DF SS MS F P**

Regression 1 0.0368 0.0368 0.464 0.566

Residual 2 0.159 0.0793

Total 3 0.195 0.0651

Normality Test (Shapiro-Wilk) Passed (P = 0.239)

Constant Variance Test (Spearman Rank Correlation): Failed (P = <0.001)

Power of performed test with alpha = 0.050: 0.067

The power of the performed test (0.067) is below the desired power of 0.800.

Less than desired power indicates you are less likely to detect a difference when one actually exists. Negative results should be interpreted cautiously.

**INH**

**Linear Regression**

**Data source:** Data 1 in Notebook1

Col 4 = 0.289 - (0.0198 * Col 1)

N = 4 Missing Observations = 1

R = 0.831 Rsqr = 0.691 Adj Rsqr = 0.536

Standard Error of Estimate = 0.006

**Coefficient Std. Error t P**

Constant 0.289 0.0641 4.508 0.046

Col 1 -0.0198 0.00935 -2.113 0.169

Analysis of Variance:

**DF SS MS F P**

Regression 1 0.000176 0.000176 4.465 0.169

Residual 2 0.0000786 0.0000393

Total 3 0.000254 0.0000847

Normality Test (Shapiro-Wilk) Passed (P = 0.667)

Constant Variance Test (Spearman Rank Correlation): Failed (P = <0.001)

Power of performed test with alpha = 0.050: 0.221

The power of the performed test (0.221) is below the desired power of 0.800.

Less than desired power indicates you are less likely to detect a difference when one actually exists. Negative results should be interpreted cautiously.

**PAS**

**Linear Regression**

**Data source:** Data 1 in Notebook1

Col 5 = 0.332 - (0.00566 * Col 1)

N = 4 Missing Observations = 1

R = 0.0338 Rsqr = 0.00114 Adj Rsqr = 0.000

Standard Error of Estimate = 0.079

**Coefficient Std. Error t P**

Constant 0.332 0.813 0.409 0.722

Col 1 -0.00566 0.119 -0.0478 0.966

Analysis of Variance:

**DF SS MS F P**

Regression 1 0.0000144 0.0000144 0.00228 0.966

Residual 2 0.0126 0.00632

Total 3 0.0127 0.00422

Normality Test (Shapiro-Wilk) Passed (P = 0.199)

Constant Variance Test (Spearman Rank Correlation): Failed (P = <0.001)

Power of performed test with alpha = 0.050: 0.027

The power of the performed test (0.027) is below the desired power of 0.800.

Less than desired power indicates you are less likely to detect a difference when one actually exists. Negative results should be interpreted cautiously.

**RIF**

**Linear Regression**

**Data source:** Data 1 in Notebook1

Col 6 = -0.0141 + (0.00469 * Col 1)

N = 4 Missing Observations = 1

R = 0.840 Rsqr = 0.706 Adj Rsqr = 0.559

Standard Error of Estimate = 0.001

**Coefficient Std. Error t P**

Constant -0.0141 0.0147 -0.962 0.437

Col 1 0.00469 0.00214 2.190 0.160

Analysis of Variance:

**DF SS MS F P**

Regression 1 0.00000992 0.00000992 4.796 0.160

Residual 2 0.00000414 0.00000207

Total 3 0.0000141 0.00000468

Normality Test (Shapiro-Wilk) Passed (P = 0.062)

Constant Variance Test (Spearman Rank Correlation): Failed (P = <0.001)

Power of performed test with alpha = 0.050: 0.230

The power of the performed test (0.230) is below the desired power of 0.800.

Less than desired power indicates you are less likely to detect a difference when one actually exists. Negative results should be interpreted cautiously.

**POA**

**Linear Regression**

**Data source:** Data 1 in Notebook1

Col 8 = -23669.466 + (3750.320 * Col 1)

N = 4 Missing Observations = 1

R = 0.984 Rsqr = 0.968 Adj Rsqr = 0.952

Standard Error of Estimate = 322.000

**Coefficient Std. Error t P**

Constant -23669.466 3292.008 -7.190 0.019

Col 1 3750.320 480.010 7.813 0.016

Analysis of Variance:

**DF SS MS F P**

Regression 1 6329206.342 6329206.342 61.043 0.016

Residual 2 207368.628 103684.314

Total 3 6536574.970 2178858.323

Normality Test (Shapiro-Wilk) Passed (P = 0.365)

Constant Variance Test (Spearman Rank Correlation): Failed (P = <0.001)

Power of performed test with alpha = 0.050: 0.674

**BEN**

**Linear Regression**

**Data source:** Data 1 in Notebook1

Col 9 = -12717.318 + (2087.566 * Col 1)

N = 4 Missing Observations = 1

R = 0.966 Rsqr = 0.932 Adj Rsqr = 0.898

Standard Error of Estimate = 266.890

**Coefficient Std. Error t P**

Constant -12717.318 2728.579 -4.661 0.043

Col 1 2087.566 397.856 5.247 0.034

Analysis of Variance:

**DF SS MS F P**

Regression 1 1961069.791 1961069.791 27.531 0.034

Residual 2 142460.515 71230.257

Total 3 2103530.306 701176.769

Normality Test (Shapiro-Wilk) Passed (P = 0.439)

Constant Variance Test (Spearman Rank Correlation): Failed (P = <0.001)

Power of performed test with alpha = 0.050: 0.525

**PIC**

**Linear Regression**

**Data source:** Data 1 in Notebook1

Col 10 = -17664.767 + (2945.462 * Col 1)

N = 4 Missing Observations = 1

R = 0.978 Rsqr = 0.957 Adj Rsqr = 0.936

Standard Error of Estimate = 294.845

**Coefficient Std. Error t P**

Constant -17664.767 3014.379 -5.860 0.028

Col 1 2945.462 439.529 6.701 0.022

Analysis of Variance:

**DF SS MS F P**

Regression 1 3904085.096 3904085.096 44.909 0.022

Residual 2 173866.908 86933.454

Total 3 4077952.004 1359317.335

Normality Test (Shapiro-Wilk) Passed (P = 0.932)

Constant Variance Test (Spearman Rank Correlation): Failed (P = <0.001)

Power of performed test with alpha = 0.050: 0.618

**SAL**

**Linear Regression**

**Data source:** Data 1 in Notebook1

Col 11 = -1894.191 + (336.442 * Col 1)

N = 4 Missing Observations = 1

R = 0.997 Rsqr = 0.994 Adj Rsqr = 0.992

Standard Error of Estimate = 11.898

**Coefficient Std. Error t P**

Constant -1894.191 121.638 -15.572 0.004

Col 1 336.442 17.736 18.969 0.003

Analysis of Variance:

**DF SS MS F P**

Regression 1 50936.996 50936.996 359.837 0.003

Residual 2 283.112 141.556

Total 3 51220.107 17073.369

Normality Test (Shapiro-Wilk) Passed (P = 0.386)

Constant Variance Test (Spearman Rank Correlation): Failed (P = <0.001)

Power of performed test with alpha = 0.050: 0.908

**CCCP**

**Linear Regression**

**Data source:** Data 1 in Notebook1

Col 12 = -73.123 + (12.391 * Col 1)

N = 4 Missing Observations = 1

R = 0.990 Rsqr = 0.981 Adj Rsqr = 0.971

Standard Error of Estimate = 0.828

**Coefficient Std. Error t P**

Constant -73.123 8.464 -8.639 0.013

Col 1 12.391 1.234 10.040 0.010

Analysis of Variance:

**DF SS MS F P**

Regression 1 69.091 69.091 100.797 0.010

Residual 2 1.371 0.685

Total 3 70.462 23.487

Normality Test (Shapiro-Wilk) Passed (P = 0.124)

Constant Variance Test (Spearman Rank Correlation): Failed (P = <0.001)

Power of performed test with alpha = 0.050: 0.757
